# miRViz: a novel webserver application to visualize and interpret microRNA datasets

**DOI:** 10.1093/nar/gkaa259

**Published:** 2020-04-22

**Authors:** Pierre Giroux, Ricky Bhajun, Stéphane Segard, Claire Picquenot, Céline Charavay, Lise Desquilles, Guillaume Pinna, Christophe Ginestier, Josiane Denis, Nadia Cherradi, Laurent Guyon

**Affiliations:** Univ. Grenoble Alpes, CEA, IRIG, Inserm, BCI, 38000 Grenoble, France; Univ. Grenoble Alpes, CEA, IRIG, Inserm, BGE, 38000 Grenoble, France; Univ. Grenoble Alpes, CEA, IRIG, Inserm, BGE, 38000 Grenoble, France; Univ. Grenoble Alpes, CEA, IRIG, Inserm, BGE, 38000 Grenoble, France; Univ. Grenoble Alpes, CEA, IRIG, Inserm, BGE, 38000 Grenoble, France; Univ. Grenoble Alpes, CEA, IRIG, Inserm, BCI, 38000 Grenoble, France; Institute for Integrative Biology of the Cell (I2BC), CEA, CNRS, University Paris-Sud, University Paris-Saclay, F-91191 Gif-sur-Yvette, France; Aix-Marseille Université, CNRS, INSERM, Institut Paoli-Calmettes, CRCM, Epithelial Stem Cells and Cancer lab, F-13273 Marseille, France; Univ. Grenoble Alpes, CEA, IRIG, Inserm, BCI, 38000 Grenoble, France; Univ. Grenoble Alpes, CEA, IRIG, Inserm, BCI, 38000 Grenoble, France; Univ. Grenoble Alpes, CEA, IRIG, Inserm, BCI, 38000 Grenoble, France; Univ. Grenoble Alpes, CEA, IRIG, Inserm, BGE, 38000 Grenoble, France

## Abstract

MicroRNAs (miRNAs) are small non-coding RNAs that are involved in the regulation of major pathways in eukaryotic cells through their binding to and repression of multiple mRNAs. With high-throughput methodologies, various outcomes can be measured that produce long lists of miRNAs that are often difficult to interpret. A common question is: after differential expression or phenotypic screening of miRNA mimics, which miRNA should be chosen for further investigation? Here, we present miRViz (http://mirviz.prabi.fr/), a webserver application designed to visualize and interpret large miRNA datasets, with no need for programming skills. MiRViz has two main goals: (i) to help biologists to raise data-driven hypotheses and (ii) to share miRNA datasets in a straightforward way through publishable quality data representation, with emphasis on relevant groups of miRNAs. MiRViz can currently handle datasets from 11 eukaryotic species. We present real-case applications of miRViz, and provide both datasets and procedures to reproduce the corresponding figures. MiRViz offers rapid identification of miRNA families, as demonstrated here for the miRNA-320 family, which is significantly exported in exosomes of colon cancer cells. We also visually highlight a group of miRNAs associated with pluripotency that is particularly active in control of a breast cancer stem-cell population in culture.

## INTRODUCTION

MicroRNAs (miRNAs) are non-coding RNAs of around 22 nucleotides that regulate protein-coding gene products at the post-transcriptional level, by directing the RNA-induced silencing complex (RISC) to its mRNA targets. Canonical binding of miRNAs corresponds to almost perfect Watson–Crick pairing of the so-called ‘seed’ sequence with its mRNA targets, often in the 3′ UTR (1). The seed sequence comprises the six nucleotides at positions 2–7 in the 5′ region of the mature miRNA. Due to the small number of nucleotides involved in this target recognition, miRNAs lack specificity, and they often have dozens to hundreds of target mRNAs. Groups of miRNAs that share the same seed sequence bind to similar sets of mRNA targets, and are thus classified as miRNA families (1).

The popularization of high-throughput technologies has generated a vast quantity and diversity of large tables of miRNAs. For example, microarrays and sequencing technologies measure miRNA expression levels under various experimental conditions, to provide data that are often converted into differential expression levels (2–4). Similarly, phenotypic high-content screening has led to large functional datasets (5). The common questions are then how to interpret these tables, and which miRNAs to select for further evaluation and validation. In the following, we define as ‘hits’ those miRNAs with high scores that indicate their potential interest. These can be differentially expressed miRNAs, highly expressed miRNAs, or miRNAs with high scores or small *P*-values.

To help in the analysis of such long lists of miRNAs, we have designed and built a free-to-use webserver to visualize and interpret miRNA datasets, entitled miRViz (http://mirviz.prabi.fr/). With miRViz, users can visualize their own and/or pre-loaded miRNA datasets on predefined miRNA networks, with various options to highlight or hide subsets of the data. These operations are accessible through the top menu on the left bandeau. The data-processing methods, such as data normalization, are not implemented in miRViz; these analyses should be conducted before using miRViz. For example, analysis for differential expression should be performed with one of the numerous available tools, such as DESeq2 (6), before visualization in miRViz.

MiRViz is designed to be as intuitive as possible. Additionally, the miRViz help file (‘Download help’, top-right) guides users, click by click, together with a downloadable video tutorial. While a few studies, including our previous study, have proposed miRNA-network approaches (7,8), to the best of our knowledge, none of them have proposed a dedicated tool for biologists to interpret miRNA datasets. There are tools to build miRNA–mRNA target networks, including miRNet (http://www.mirnet.ca/), although the users have to previously identify miRNAs of interest from their datasets (9). MiRViz and miRNet can thus be used in sequence. Bracken *et al.* reviewed the websites for enrichment analysis (10). These websites are also complementary to miRViz, and can be used with the same datasets. For example, TAM (http://www.lirmed.com/tam2/Home/) and miEAA (https://ccb-compute2.cs.uni-saarland.de/mieaa_tool/) perform ontology enrichment at the level of miRNAs, and miRPath (http://snf-515788.vm.okeanos.grnet.gr/) and miRPathDB (https://mpd.bioinf.uni-sb.de/) do so at the level of the mRNA targets (11–14).

This paper is organized as follows. In the following section, we present the different miRNA networks and the preloaded datasets. We then present the webserver functionalities, and provide practical examples to highlight the strengths of miRViz for experimental data analysis. Importantly, in the Supplementary Materials we provide all of the necessary datasets and step-by-step procedures to reproduce the figures shown here with miRViz.

## MATERIALS AND METHODS

### Implementation

MiRViz is composed of a JavaScript front-end server and a Java back-end server. While the best user experience is provided when running miRViz on wide screens with high resolution, it still remains of interest with lower resolution, where zooming out may sometimes be necessary to avoid overlapping components (for more details, see the Troubleshooting section of the downloadable mirviz-help.pdf). User datasets are not uploaded to the server but are safely loaded into the local browser. MiRViz is freely available without login requirements.

### MicroRNA networks

MiRViz is built around predefined miRNA networks, in which each node is represented by a circle that corresponds to a unique mature miRNA. In its 2020 version, miRViz can be used to visualize miRNA data from 11 different species, including human (hsa), mouse (mmu), *Caenorhabditis elegans* (cel) and Drosophila (dme). The architecture of the webserver was designed to easily add new networks and new species in the future.

In miRViz, we propose eight different predefined networks. Each of the networks has its own rules for the connection of the miRNA nodes. The first network used by miRViz, ‘Seed2_7’, allows direct visualization of miRNA families by connecting pairs of miRNA nodes that share the same seed sequence. The two miRViz networks entitled ‘Genomic_Distance’ connect neighboring miRNA genes on the genome. The ‘2k’ version links neighboring miRNAs if they are closer than 2 kilobases (kb), to visually identify polycistronic miRNA clusters (15). The ‘50k’ version has a less stringent threshold of 50 kb, to account for large genomic reorganizations, as can be encountered in tumors and as demonstrated in an example below. Three co-regulation networks connect miRNA nodes that share common mRNA targets (7). More precisely, in ‘Diana50’, ‘TargetScan54’ and ‘DianaTarBase50’, the miRNA nodes are connected if they share more than 50%, 54% and 50% common mRNA targets, respectively, as predicted by Diana MicroT v3 (16) or Target-Scan v6.2 (17), or by experimental validation and gathering in Diana-TarBase v8 (18). We added two simplified networks with fewer nodes to ease visualization and interpretation: ‘Genomic_Distance_50k_clusters_3+’ which only keeps the 820 miRNAs that are in clusters of size 3 or more, and ‘TargetScan54_degree_10+’ which keeps the same layout of ‘TargetScan54’ but removing the nodes connected to 9 or fewer nodes. In the Supplementary Materials, we provide the exact formulae and justifications of the rules that underlie the connections of the miRNA nodes in each of the networks.

### Pre-loaded datasets

Three miRNA tables are pre-loaded in miRViz to ease comparisons with user datasets: ‘hsa_miRNAmine_cells’, ‘hsa_miRNAmine_tissues’ and ‘hsa_TissueAtlas’. For the two first datasets, the data were gathered from miRNAmine (3). For the third dataset, the data were gathered from TissueAtlas (4). MiRNA expression was transformed into log_2_ scales, and then averaged across all of the experiments performed under the same conditions (the number of different experiments used to average is indicated in brackets). Expression on a log_2_ scale spans from 0 to 20.

### Experimental microRNA datasets

We have used various public miRNA expression datasets in the practical examples provided (from both microarrays and sequencing) to highlight miRViz strength (19,20). We have also used an in-house functional miRNA screening dataset (21). The datasets are provided as Supplementary Tables and the data pre-processings are detailed in the Supplementary Materials. Briefly, the first dataset relates to miRNA sequencing, from the public identifier SRA106214. It provides miRNA expression levels in colon cancer cell line LIM1863, and in their secreted small vesicles, together with differential expression between vesicles content and the parental cells (Supplementary Table S1). The second dataset provides the prognostic value for overall survival of patients with adrenocortical carcinoma, for each individual miRNA, expressed in the log_10_ of the *P*-value obtained after log-rank test (Supplementary Table S2). The third dataset provides the differential expression between human embryonic stem cell and differentiated cells from the public dataset GSE14473 (Supplementary Table S3). The fourth dataset provides the change of the percentage of breast cancer stem cells after transfection of individual miRNA mimics (Supplementary Table S4). The last dataset presents the enrichment of two selected gene ontologies among predicted mRNA targets for each individual miRNA (‘regulation of gene expression’ and ‘small-GTPase-mediated signal transduction’, Supplementary Table S5).

### Statistical tests

MiRViz provides visualization of the aggregation of miRNAs on various networks, in particular inside families (Seed2_7 network) or genomic clusters (Genomic_Distance networks). To assess how significant miRNA hits are aggregated in a given family or cluster, a ‘local’ statistical test can be performed. Let *p* denote the proportion of hits on the network (*p = n_hits / n_nodes*, with *n_hits* the number of hits, and *n_nodes* the number of nodes with miRNA measurement in the network*)*, and *n_cluster* the number of nodes in the cluster/family of interest with miRNA measurement (e.g. expressed miRNAs for differential expression measurements). Under the null hypothesis H_0_, the number of hits in this cluster/family is given by the binomial distribution, and the *P*-v*alue* is the probability under H_0_ to get equal or more hits in the cluster/family of interest. To assess how significant miRNA hits are connected in a given network, we also proposed a ‘global’ statistical test, in which the number of hit pairs (i.e. an edge connecting two miRNA hit nodes) are first counted. Then, to evaluate the null hypothesis H_0_, the number of hit pairs is counted after a hit randomization procedure in which an miRNA is randomly designated a hit, while keeping constant the total hit number. The global *P*-value is estimated as the proportion of randomized trials for which the measured number of hit pairs is below the randomized one.

## RESULTS AND DISCUSSION

### MiRViz highlights the selective export of the miR-320 family into colon cancer cell exosomes

**Figure 1.**
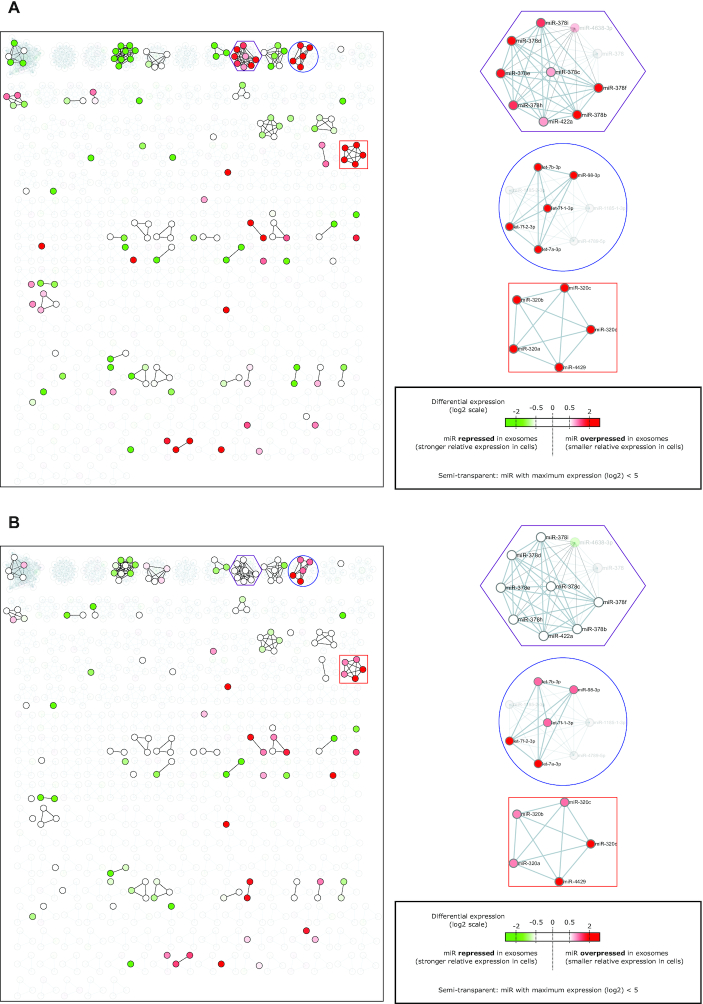

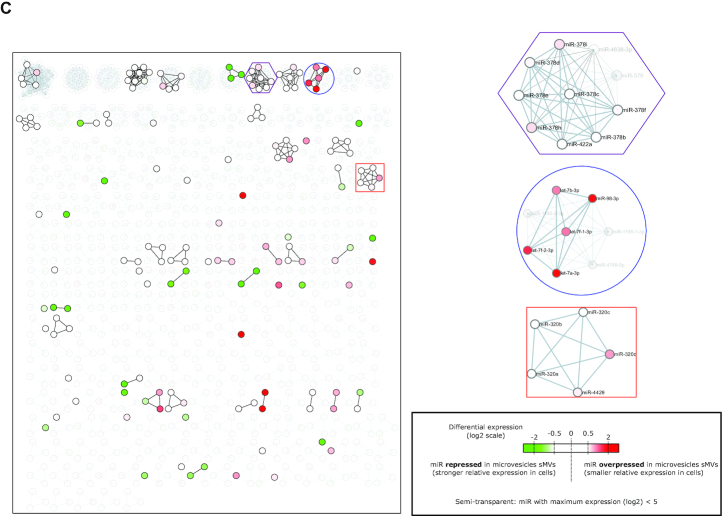
(A–C) Color scale representation of the differentially expressed miRNAs between the exosomes or microvesicles and the parental LIM1863 cells overlaid on the left hand side of the Seed2_7 network of miRViz. MiRNA families naturally appear from the largest to the smallest. Red and green nodes correspond to miRNAs overexpressed and repressed in vesicles, respectively. Three interesting clusters are zoomed in on at the right side: The miRNA cluster in the red square corresponds to the miR-320 family, the purple hexagon corresponds to the miR-378/422a family, and the blue circle to the let-7-3p/miR-98-3p family. Nodes corresponding to miRNAs not expressed in this cell type were set to semi-transparent. (**A**) MicroRNAs in A33 exosomes derived from colon cancer cells *versus* parental cells. (**B**) MicroRNAs in EpCAM exosomes derived from colon cancer cells versus parental cells. (**C**) MicroRNAs in shed microvesicles versus parental cells.

The first network used by miRViz, Seed2_7, allows direct visualization of miRNA families by connecting pairs of miRNA nodes that share the same seed sequence. The Seed2_7 networks can be visualized through miRViz for all of the 11 species proposed. As a demonstration of how miRViz can be used to interpret miRNA expression datasets, a few click tutorials are provided in the help file (top-right button on the website), which shows how to visualize tissue-specific miRNAs. Here, we propose a complementary example using the publicly available miR-seq dataset that profiles the LIM1863 colon cancer cell line and three different sorts of extracellular vesicles isolated from the culture supernatants (19). Figure 1 shows the differentially expressed miRNA families in two types of immunoaffinity-isolated exosomes (i.e. A33 in Figure 1A; EpCAM in Figure 1B) and shed microvesicles (Figure 1C). MiRNAs with lower and higher expression in vesicles compared to cells are colored in green and red, respectively. The nodes that correspond to the miRNAs with very low expression under all of the conditions is set as semi-transparent. Interestingly, miRNAs tend to have similar differential expression in each family, which is highlighted by miRViz showing uniform colors in each family. Indeed, when defining an miRNA with high differential expression (log_2_ fold change > 1) as a hit, miRViz shows that hits significantly aggregate in miRNA families in all three sort of vesicles (global *P*-value < 10^−5^, Supplementary Materials section 6). We can thus hypothesize that there is an active export of selective family members through exosomes, in accordance with active export shown in other experimental cases (22,23). MiRViz can quickly identify these families, and provide a way to share the result. The hsa-miR-378/422a family is exported specifically in immunoaffinity-isolated A33 exosomes (*P*-value = 0.008; paired Wilcoxon test; Figure 1A). The hsa-miR-320 family is significantly exported in both sorts of exosomes (*P*-value = 0.002; paired Wilcoxon test; Figure 1A, B). This export is clear only for exosomes (i.e. not for small vesicles), and stronger for the A33 exosomes. We have confirmed these data by independent RT-qPCR measurements (Supplementary Figure S1). Finally, all of the five expressed members of the hsa-let-7-3p/miR-98-3p family are significantly exported in all of the three types of extracellular vesicles (*P*-value = 6 × 10^−5^; paired Wilcoxon test; Figure 1). In addition to overall aggregation behavior of miRNA hits, one can ask how significant hits are aggregated in a given family. All the three families described above for which the differential expression was significant, showed also a significant segregation (local *P*-value < 0.002, Supplementary Materials section 6).

### High expression of miRNAs of the Xq27.3 cluster is predictive of better prognosis in adrenocortical carcinomas

**Figure 2.**
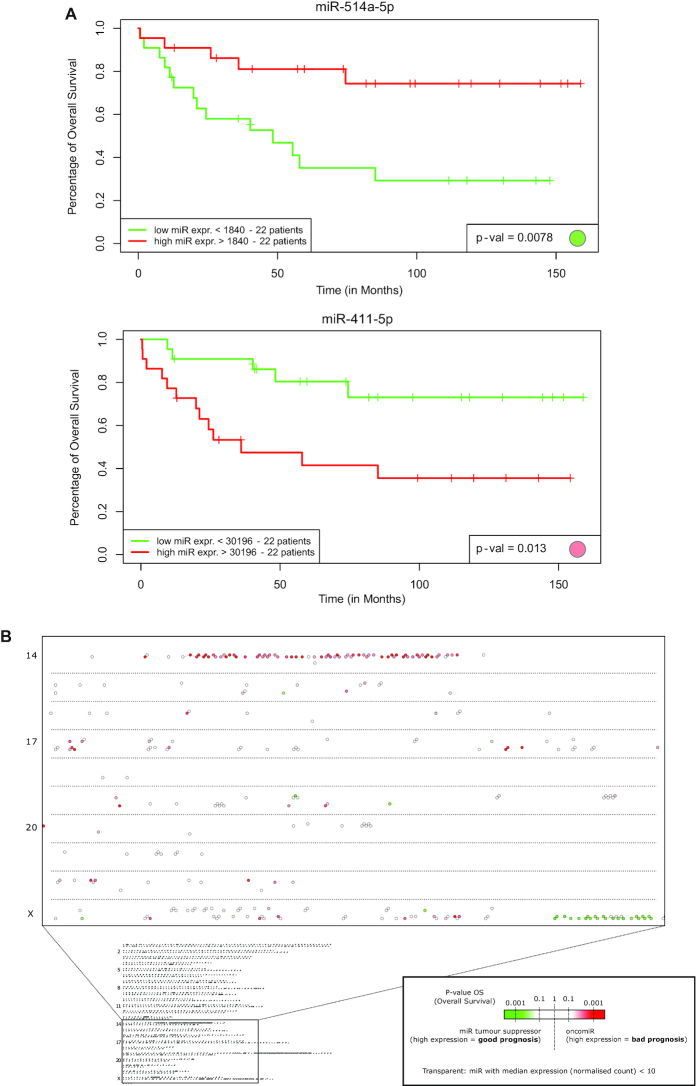

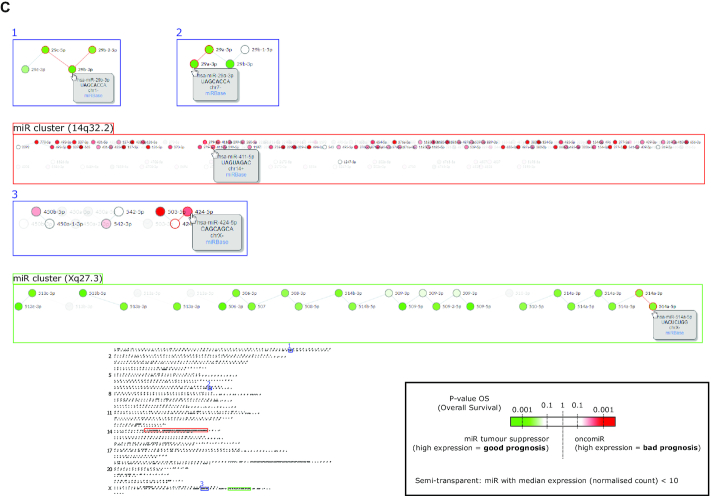
Prognostic potential of miRNAs for overall survival of patients with adrenocortical carcinoma. (**A**) Kaplan–Meier curves for miR-514a-5p (top) and miR-411-5p (bottom). The *P*-value calculated with the log-rank test is indicated at the bottom right of each plot, together with the node colored accordingly using the color scale chosen in (B, C). (**B**) Prognostic value of individual miRNAs overlaid on the Genomic Distance 50k network. Bottom: View of the whole Genomic_Distance_50k network. The square correspond to the zoomed in area displayed above. Chromosomes are organized from top to bottom (1–22, X, Y). MiRNAs for which high expression correlates with poor prognosis are highlighted in red. Good prognosis miRNAs are represented in green. MiRNAs with low expression are set as transparent. (**C**) MiRViz screen shots of interesting areas that show miRNA names and the action of the mouse pointer on a given node. The squares on the full network below correspond to the interesting areas. MiRNAs with low expression are set as semi-transparent. A few small clusters of miRNAs with high differential expression are highlighted (blue squares): Clusters 1 and 2 correspond to miR-29 family located on chromosomes 1 and 7, and cluster 3 correspond to miR-503-5p/424-5p located on chromosome X. The two major clusters in green and red squares (i.e. Xq27, 14q32) of 95 and 197 kilobases, respectively, show groups of miRNAs associated with good and poor prognosis, respectively.

Both Genomic_Distance networks can be visualized with miRViz for all of the 11 species. To demonstrate the interest of the Genomic_Distance_50k network in the context of cancer, we reanalyzed the public data from Assie *et al.* (24), which contains both the miRNome of tumor samples from patients diagnosed with adrenocortical carcinoma and their overall survival (OS) information. For each miRNA, the patients were separated into two groups of equal size, which depended on the miRNA quantification, and a *P*-value was calculated on the OS after log-rank tests. A hit is defined here as an expressed miRNA (median expression among patients > 10 reads) for which the *P*-value is <10^−2^. Figure 2A shows the Kaplan-Meier curves of two microRNAs of interest, together with the *P*-value of the log-rank test, and the node colored according to the *P*-value. Figure 2B shows the *P*-value in a log10 scale overlaid onto Genomic_Distance_50k network, zoomed in on chromosomes 14 to X, with a green gradient for good prognosis miRNAs (miRNAs for which high expression is of good prognosis for the patient), and a red gradient for poor prognosis miRNAs. Two large clusters show up in miRViz (Figure 2B, C):

- cluster 14q32.2 (spanning 197 kb) that is predictive of poor prognosis, i.e. patients who show high expression of the miRNAs of the cluster are associated with shorter OS (24 hits out of 48 expressed miRNAs, *P*-value = 6.1 × 10^−7^);
- cluster X27q3 (spanning 95 kb) that is predictive of good prognosis, i.e. patients who show high expression of these miRNAs are associated with longer OS (9 hits out of 13 expressed miRNAs, *P*-value = 6.7 × 10^−7^).

While both clusters were described in the original publication (24), miRViz proposes a rapid method to easily identify such clusters and a way to visualize the data. Additionally, in Figure 2C three clusters are highlighted in blue: two clusters of the hsa-miR-29 family, located in 1q32.2 and 7q32.3, and associated with good prognosis (three hits out of four in each cluster, *P*-value = 4.1 × 10^−3^); and the hsa-miR-450b-5p/503-5p/424-5p cluster, located in Xq26.3 and associated with adverse prognosis (two hits out of seven expressed miRNAs, *P*-value = 0.37). The latter is not significantly enriched, as it is probable to find a few miRNA hits out of seven just by chance when there are so many hits (in this case 44 hits out of 241, most of the hits are in the 14q32 cluster). It is interesting to note that the mature miRNAs from the -3p strand of the miR-29 families that are transcribed from both chromosomes 1 and 7 share the same seed, AGCACC, which suggests redundancy.

### MiRViz visually identifies the miR-302/519 stem-cell family in the regulation of breast cancer stem cell equilibrium

**Figure 3.**
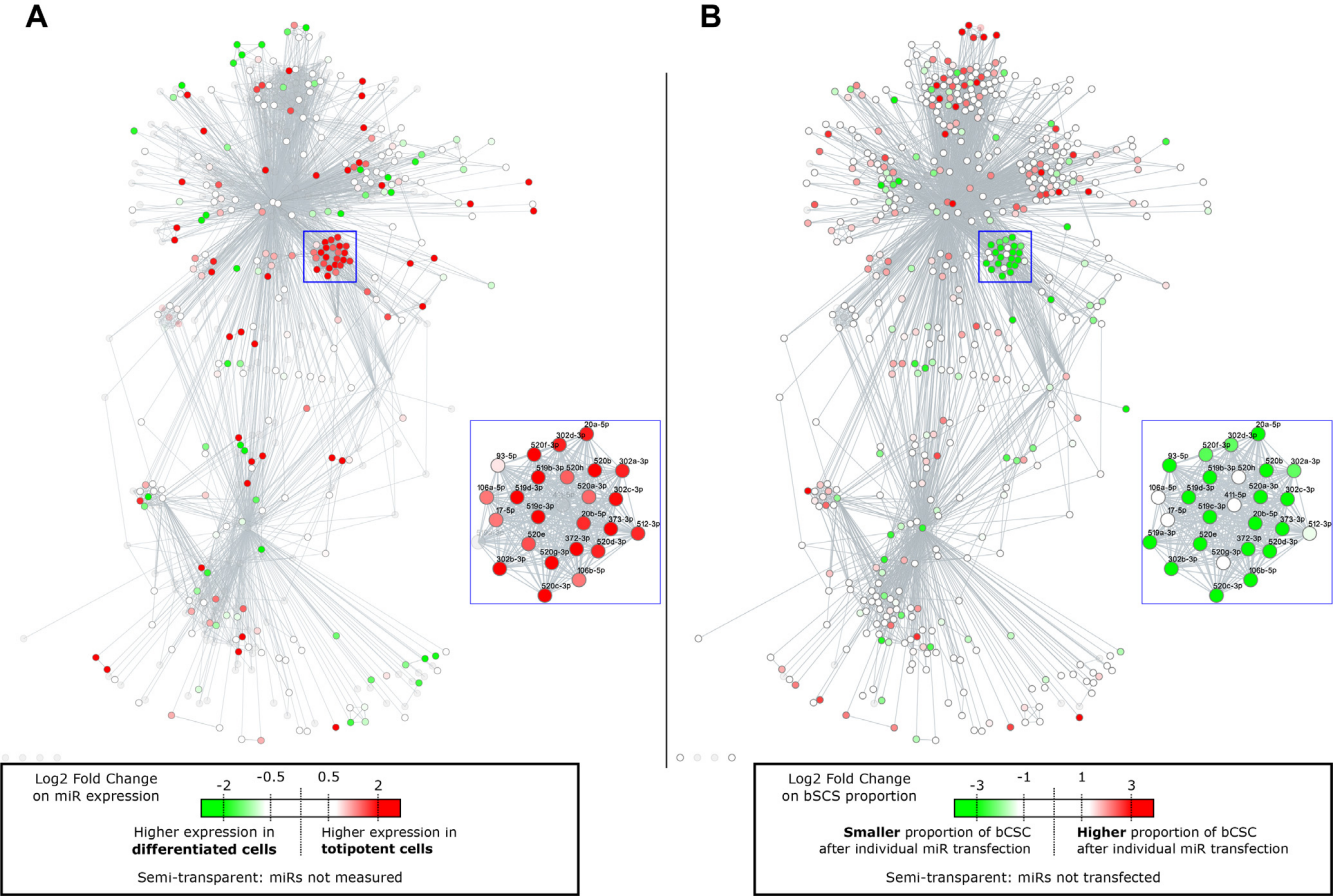
(**A**) Differential expression of miRNAs from cells grown in totipotent medium versus differentiation medium, as obtained from the GSE14473 public dataset (20), overlaid on the Diana50 network. MiRNA nodes in red correspond to miRNAs overexpressed in totipotent cells. (**B**) Changes in the bCSC relative proportions after miRNA overexpression. MiRNA nodes in green correspond to miRNAs for which overexpression leads to decreased proportions of bCSCs. (**A, B**). Blue squares show the clusters described in the main text, which are zoomed in on at the side of the whole network.

As proof of purpose for the Diana50 network, Figure 3A shows the differential expression of miRNAs in human embryonic stem cells that were cultured under two different conditions that favor either pluripotency or differentiation. Here, a ‘stem cell’ miRNA cluster clearly shows up in red, which highlights the overexpressed miRNAs in the pluripotent stem cells. Most of these miRNAs have already been hypothesized to cooperatively regulate pluripotency (25). The group comprises miR-17/20/93/106/302/../519/520 with shifted seed sequences (AAAGUG, AAGUGC, AGUGCU), and miR-411 with seed sequence AGUAGA.

Figure 3B represents a functional screening dataset where we measured the relative levels of breast cancer stem cells (bCSCs) in a human breast adenocarcinoma cell line (SUM159 cells) upon systematic and individual overexpression of miRNAs (21). The green (resp. red) nodes represent miRNAs that upon overexpression lead to smaller (resp. higher) bCSC proportions. To determine the efficiency of miRViz to compare different datasets and the possibility that it can raise biological questions of interest, we can compare Figure 3A and B. Here, the miRViz representation highlights the redundant action of the ‘miRNA stem cell’ cluster on the balance of the bCSC phenotype. It is, however, surprising that miRNAs for which expression was correlated with pluripotency in normal cells (Figure 3A, in red) indeed lead to decreased proportions of bCSCs when overexpressed (Figure 3B, in green). This suggests that the fine-tuning of this specific group of miRNAs might have an important and yet unknown role in the maintenance of the ‘stem’ state of normal and cancer cells. Interestingly, miRViz identifies this group of miRNAs, and suggests target gene redundancy, which might explain why their individual knock-downs in separate experiments (data not shown) had little or no effects on the bCSC equilibrium. To assess these hypotheses, additional experiments are necessary. Results obtained with miRViz suggest that these miRNAs needs to be collectively studied, and that their simultaneous knock-down may help to restore the expression of the target genes responsible for the stem-cell features.

### Diana50 and TargetScan54 structures are correlated with biological functions

**Figure 4.**
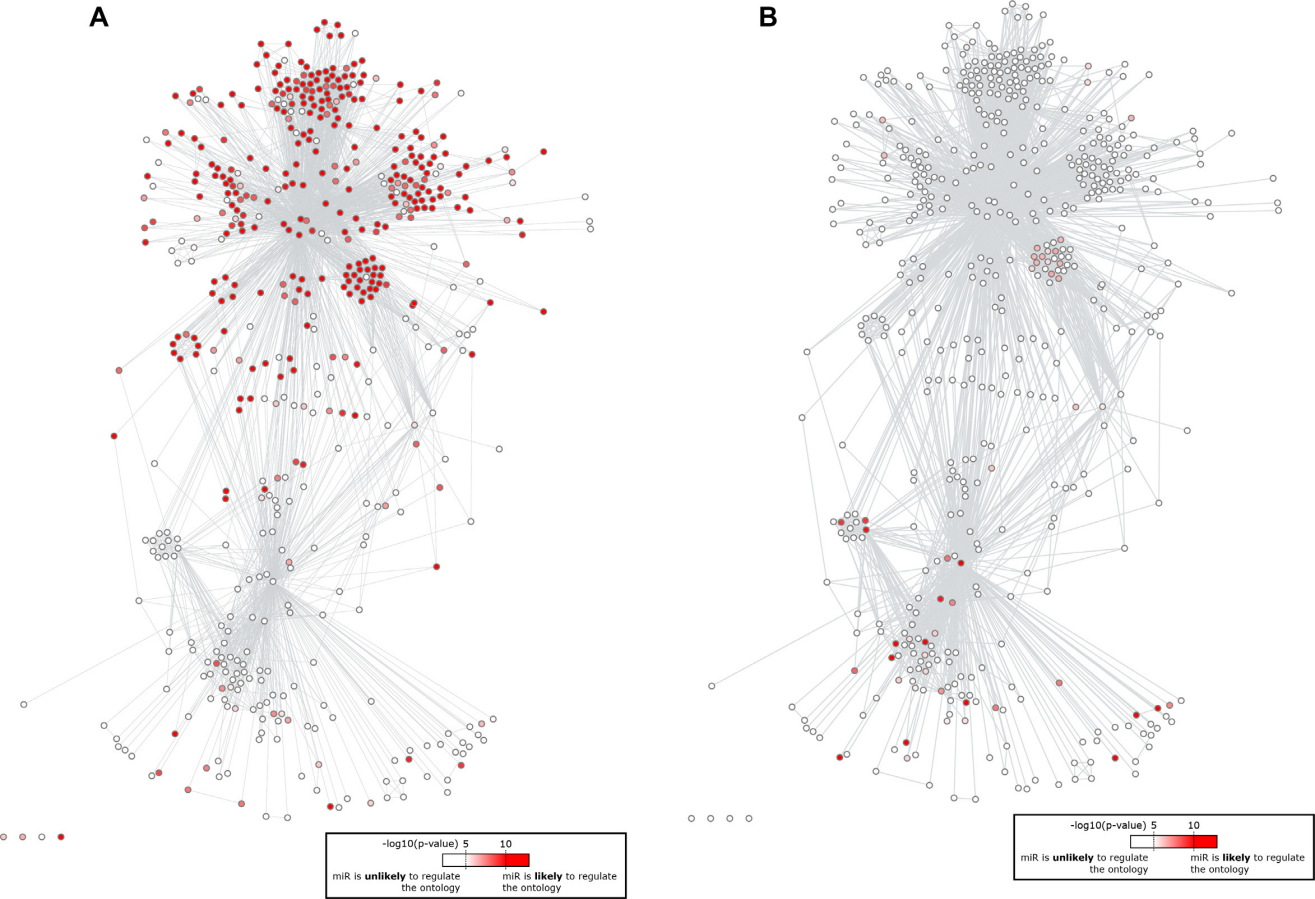
Gene ontology enrichment for predicted targets of individual miRNAs overlaid on top of the Diana50 network. Red nodes correspond to miRNAs predicted to regulate many protein coding genes known to participate in the following ontologies: (**A**) GO:0010468 (regulation of gene expression); (**B**) GO:0007264 (small-GTPase-mediated signal transduction).

To show the link between network organization and miRNA functions, we performed gene ontology enrichment on the predicted mRNA targets for each individual miRNA. For a given ontology and miRNA, the small *P*-values (typically <10^−5^) suggest that the miRNA regulates the corresponding function under certain cellular conditions. Figure 4 and Supplementary Figure S2 show that miRNAs that are assumed to regulate a given ontology (i.e. pathway or function) are not randomly spread out in the networks. Supporting our previous study (7), the Diana50 and TargetScan54 networks are structured in two parts. The upper part of both networks contains miRNAs that are almost all predicted to regulate gene expression, together with the two more central subnetworks of let-7 and miR-17/93 (Figure 4A, Supplementary Figure S2A). The lower parts of both of these networks contain many miRNAs that are predicted to regulate signal transduction through small GTPases (Figures 4A, Supplementary Figure S2B). Altogether, these observations show that the Diana50 and TargetScan54 structures correlate with biological predictions, and that the positions of the hits in the networks inform the users of putative regulated pathways, e.g. miRNA hits in the upper part might be important regulators of gene expression.

### Which network for which dataset, and further validations

An important question is: which network should be used for a given dataset? The simple answer is ‘all’. We suggest to use all the networks, starting with small ones (Seed2_7, Diana50 and Genomic_Distance_50k_clusters_3+ for human). We suggest then to visually identify enrichment of highly connected groups of miRNAs. In our experience, we often find enrichment in the Seed2_7 and/or Genomic_Distance networks. Yet, the most appropriate network to explore a specific dataset depends on the biological question to answer. When focusing on mRNA targets repressed by miRNAs, the Seed2_7 (and in a second time the co-regulation networks: Diana50, DianaTarBase50 and TargetScan54) should be used. When trying to identify polycistrons, Genomic_Distance_2k shows co-expressed miRNAs in a given neighborhood on the genome. The 50k version is more dedicated for large genomic reorganizations, as found in cancer. To note, even if miRViz is particularly useful for raw expression and differential expression datasets, any large miRNA dataset with numerical scores can benefit from miRViz. Figures 2, 4 described above present practical examples with *P*-values and phenotypic scores.

Another important question is: what to do with miRViz results? First, the mapping itself is interesting, and miRViz provides an export function to show a given enrichment in a figure. Second, it guides the validation steps. When investigating the phenotypic role of a given miRNA, a practical experiment consists in knocking-down (or over expressing) this miRNA and measure the phenotypic outcome. If many miRNAs of a given family are co-expressed, modulating one miRNA out of the whole family may lead to no or minimal phenotypic effect, as the other miRNAs from the family may still repress the mRNA targets. A suggestion would be to modulate the whole family, which is also true for groups of highly connected miRNAs in co-regulation networks, such as for the miR-302/519 stem-cell family detailed above.

## CONCLUSION

We propose that the webserver application miRViz can be used to visualize numerical miRNA datasets. We have illustrated the results that can be obtained for miRViz through various examples, including miRNA expression and functional screening datasets. For miRNA profiling, the network-based visualization proposed here provides clear ways to present datasets that are complementary to volcano plots for expression data. In particular, the Seed2_7 network allows rapid identification of hit miRNA families, and quickly identifies miRNA redundancies.

## Supplementary Material

gkaa259_Supplemental_Files
