# Supplementary material for: miRViz: a novel webserver application to visualize and interpret microRNA datasets
Source: Nucleic Acids Res. 2020 Apr 22;48(W1):W252–61. doi: 10.1093/nar/gkaa259 (PMC7319447; doi:10.1093/nar/gkaa259)
Supplement: gkaa259_Supplemental_Files [file gkaa259_supplemental_files.zip › 20-03-17_LGuyon_RevisedSUPPL_miRViz_NAR2_vf.pdf]

Supplementary Materials

This Supplementary file is organized as follows. Section 1 presents the 'Seed2_7' network. Section 2 illustrates the two 'Genomic_Distance' networks. Section 3 presents the three co-regulation networks ('Diana50', 'TargetScan54', 'DianaTarBase50'). The datasets used throughout this study are presented in Section 4, and the annotated step-by-step procedures used to produce the Figures shown in this article are described in Section 5. Supplementary Tables 1-5 correspond to Figures 1-5, and allow their easy reproduction with miRViz. Section 6 provides detailed p-value results related to Figure 1.

1. Description of the Seed2_7 network 1

2. Description of the Genomic_Distance networks 2

3. The co-regulation networks: Diana50, TargetScan54, and DianaTarBase50 2

a) Description of the Diana50 and TargetScan54 networks 2

b) Description of the DianaTarBase50 network 3

4. Materials and Methods 3

a) Webserver implementation 3

b) Large-scale datasets used in the Figures 3

c) RT-qPCR validation experiments 4

d) Statistical tests 5

5. Step-by-step procedure to reproduce the Figures 6

a) Figure 1: Differential expression and miRNA families 6

b) Figure 2: Survival data and genomic positions 7

c) Figure 3A: Differential expression and Diana50 8

d) Figure 3B: Functional score and Diana50 8

e) Figure 4 and Supplementary Figure 2: Ontology enrichment and co-regulation networks 9

f) Generating high-quality images 9

6. Statistical tests results 9

a) Local statistical tests 9

b) Global statistical tests 10

References 11

Legends for Supplementary Figures 12

1. Description of the Seed2_7 network

For the networks proposed in miRViz, each node is represented by a circle that corresponds to a unique mature miRNA. The Seed2_7 networks can be visualized through the miRViz website (http://mirviz.prabi.fr/) for all of the 11 species proposed. To build this network, all of the miRNAs were retrieved from the miRBase v22.1 database[1] from a given species, and two nodes were connected if the corresponding miRNAs shared the same seed sequence, as classically defined as nucleotides 2 to 7 from the 5'-end of the mature miRNA. Seed2_7 then displays groups of interconnected nodes, which are families of miRNAs that are defined by their common seed sequence.

Due to the importance of the seed sequence for mRNA target recognition[2], all of the miRNAs from a given family are believed to share biological functions. Co-expression of miRNAs from the same family thus corresponds to functional redundancy.

To construct the network, the families were ranked from the largest to the smallest, while only considering families of two nodes or more. Individual miRNA nodes were removed; *i.e.,* the mature miRNAs that did not share their seed sequence with others were removed. In human, the Seed2_7 network contains 1,712 miRNAs, and the biggest family, which encompasses 27 nodes, corresponds to the hsa-miR-548-5p/559 family with seed sequence AAAGUA. In mouse, the network contains 1,133 mature miRNA nodes, and the biggest family, which contains 14 nodes, corresponds to the mmu-let-7-3p/miR-98-3p family of the seed sequence UAUACA. On the website, moving the mouse pointer onto a given node provides information on the corresponding mature miRNA: official name, nucleotide sequence at positions 1-8 from the 5' end (with the seed sequence in bold), chromosome location, and a link to the corresponding miRNA in miRBase[1], http://mirbase.org/.

2. Description of the Genomic_Distance networks

Both Genomic_Distance networks can be visualized with the miRViz website (http://mirviz.prabi.fr/) for all of the 11 species. To build the networks, we also retrieved all of the miRNAs from the miRBase v22.1 database[1] for a given species, together with their genomic positions. We connected two miRNA nodes to their nearest neighbor if they were closer than a fixed distance apart on the genome. The distance between two connected nodes in pixels is proportional to the logarithm of the chromosomic distance in nucleotides. We gathered 2,883 mature miRNAs for both networks in human. Chromosomes are organized from top to bottom (in human, 1 to 22; X, Y). We fixed the distance threshold at 2 kilobases (kb) for the 'Genomic_Distance_2k' network, to visually identify polycistronic miRNA clusters[3]. The 2k human network contains 1,146 links (i.e., pairs of connected nodes). Genomic_Distance networks naturally display groups of connected miRNAs, called clusters, which can correspond to polycistrons. Two miRNA nodes separated by more than 2 kb can be localized in the same cluster step by step. For example, the biggest human cluster contains 26 mature miRNAs, from hsa-miR-376c-5p to miR-655-3p, and it is 10 kb long, although each pair of miRNAs is closer than 2 kb.

The 'Genomic_Distance_50k' is defined with a threshold distance of 50 kb, which leads to larger clusters. It is designed to analyze co-expressed miRNAs following large genomic reorganization events, as encountered in cancers. The largest cluster contains 89 mature miRNAs for the 19q13.4 chromosome location. It spans 122 kb, from hsa-miR-512-5p to miR-373-5p. This primate-specific cluster is known as C19MC[4].

3. The co-regulation networks: Diana50, TargetScan54, and DianaTarBase50

a) Description of the Diana50 and TargetScan54 networks

Both 'Diana50', and 'TargetScan54' can be visualized with miRViz, as in **Figure 3** and **Supplementary Figure 2,** respectively. For these networks, we connected the miRNA nodes if they shared >50% or >54%, respectively, common mRNA targets, as predicted by Diana MicroT v3[5] or Target-Scan v6.2[6]. The metric chosen was meet/min[7]: the percentage of common targets between two miRNAs is defined as the number of common targets divided by the minimum number of targets between the two miRNAs. We have previously described both networks[7], and in particular, the choice of the thresholds. Briefly, these thresholds of 50% and 54% maximized the

‘betweenness centrality’ measurements of each network, and so reveal miRNA communities. ‘TargetScan54’ contains 1,530 nodes, each of which corresponds to a mature miRNA. ‘Diana50’ comprises 539 nodes, and gathers fewer but more commonly found miRNAs, and is more easily viewable. The networks layout was performed using Cytoscape with the unweighted spring embedded layout[7,8]. Nodes are then closer when they form dense clusters of interconnected nodes. However, these clusters were too dense, with overlapping nodes. To facilitate visualization of these clusters, and to better highlight them, we applied the layout again on these clusters separately, and pulled them to the side of the networks. Dense clusters correspond to large groups of miRNAs that share many predicted mRNA targets. As in the example described in the main text, co-expression of the miRNAs of a given cluster suggests redundancy in mRNA repression. Thus, functional validation should be performed by simultaneously knocking-down co-expressed miRNAs of a cluster, as individual miRNA knock-down might lead to only subtle phenotypic effects, if any. It is well known that mRNA target prediction algorithms contain false positives[9]. We previously showed that the network structures of Diana50 and TargetScan54 are anyway robust to the change of prediction algorithm despite distinct individual predictions[7].

b) Description of the DianaTarBase50 network

Diana TarBase gathers experimentally verified pairs of miRNA-mRNA targets[10]. While containing pairs of high confidence interactions, this database has the opposite disadvantage of missing many real interactions due to partial knowledge that are false negatives. As the human species is the most studied, we proposed the ‘DianaTarBase50’ network in miRViz. To define the network, we gathered all of the 432,361 direct miRNA-mRNA interactions of Diana TarBase, and connected two miRNA nodes if they shared more than 50% of common mRNA targets with the same meet/min formulae as for Diana50 and TargetScan54 described above. We only kept miRNAs for which at least 10 experimentally verified targets were known.

4. Materials and Methods

a) Webserver implementation

The names of all of the libraries used are listed at the end of the help file. The main library is D3.js for graphical visualization. The source code is available under the Open Source CECILL-B license in the download section.

b) Large-scale datasets used in the Figures

All of the data described below were analyzed with R version 3.4[11]. The processed datasets are provided in Supplementary Materials in csv format, directly reusable in miRViz to reproduce the Figures provided here. We detail in Section 5 all of the steps to do this.

MiRNA sequencing of small vesicles and colon cancer cells

We used colon cancer cells and the exosome miRNA sequencing public dataset SRA106214 produced by Ji and coworkers[12]. The raw data were filtered using FastQC v0.72 (http://www.bioinformatics.babraham.ac.uk/projects/fastqc/) and Trim Galore! v0.4.3.1 (https://www.bioinformatics.babraham.ac.uk/projects/trim_galore/), and then aligned against the miRBase v22 sequence list[1] using Bowtie1 v1.2.0[13], with the read counts normalized using DESeq2 v2.11.40.3 [14]. Differential expression is expressed in the log2 scale (log2[miR_count(Exo)/miR_count(Cell)]) in **Supplementary Table 1**.

Adrenocortical cancer survival data

MiRNA sequencing data from individual patient tumors diagnosed as adrenocortical carcinoma were retrieved from the GSE49279 public dataset. Clinical information, including follow-up and overall survival (OS) of the same 45 patients, were retrieved from the Supplementary Materials of Assie *et al.*[15]. For each miRNA, the population of patients was separated into two equal groups of 22 patients according to the median expression of the miRNA. P-values were calculated with log rank tests for OS with the R package 'survival' version 2.44. A score was defined by log10 transformation of the p-value, and a minus sign was added depending on the prognosis for patients with high expression of the miRNA (**Supplementary Table 2**). For example, miR-29a-3p has a score of -3.3, which corresponded to a p-value of 5.5×10^{-4}, with its high expression correlated with good patient prognosis. The node corresponding to this miRNA is pure green in **Figure 2**.

MiRNA expression with microarrays in human embryonic stem cells

The miRNA expression in human embryonic stem cells originates from the public GSE14473 dataset[16]. In this study, 10 different cell lines were separately cultured under conditions to either maintain an undifferentiated state or to promote undirected differentiation. For each miRNA, we took the median among all of the cell lines of the log2 fold-change (LFC) between the undifferentiated and differentiated states (**Supplementary Table 3**).

High-content screening of breast cancer stem cell equilibrium upon miRNA overexpression

To demonstrate the miRViz use for high-content screening datasets, we used an in-house dataset described earlier[17]. Briefly, after the individual transfection of miRNA mimics from a human miRNome-wide library, high-content screening was used to measure the proportion of breast cancer stem cells (ALDEFLUOR-positive cells) in LFC compared to the control (**Supplementary Table 4**). For example, hsa-miR-512-3p has a LFC of -1.1, which means that there were almost half the bCSCs after miR-512-3p transfection compared to control ($2^{-1.1} = 0.46$). This miRNA is represented in light green in **Figure 3B**.

Gene ontology enrichment of mRNA targets of individual miRNAs

For each mature miRNA, the pool of predicted mRNA targets by TargetScan v6.2[18] was retrieved. Gene ontology enrichment was performed on each pool, which provided a p-value for each miRNA and ontology. The p-values were expressed in –log10 scale: a value of 12 (shown in red in **Figure 4** and **Supplementary Figure 2**) corresponds to $p = 10^{-12}$ (**Supplementary Table 5**). Ontologies were retrieved using the R package GO.db version 3.1[19], and the correspondence between gene ID databases was performed with biomaRt R package version 2.26[20]. Fisher exact tests were performed to determine the p-values.

c) RT-qPCR validation experiments

The colorectal-cancer-derived LIM1863 cell line was purchased from Public Health England (Salisbury, UK). The cells were cultured in RPMI 1640 medium containing 2 mM L-glutamine, 25 mM HEPES, 10% fetal calf serum, 0.6 µg/mL insulin, 1 µg/mL hydrocortisone and 10 µM 1-thioglycerol. The cells were grown as floating organoids in a humidified incubator at 37 °C in a 5% CO_2–95% air atmosphere.

Exosomes were prepared using differential centrifugation of conditioned medium collected from LIM1863 cells. Successive centrifugations at increasing speeds were performed to eliminate dead cells and large cell debris (10 min at 300× *g*; 10 min at 2,000× *g*). The supernatant was centrifuged for 30 min at 10,000x *g* at 4 °C, to pellet the microvesicles. The final supernatant was filtered through a Millipore Stericup filtration system (pore size, 0.22 µm), and then ultracentrifuged at 100,000× *g* for 180 min to pellet the exosomes. The pellet was washed with 8 mL phosphate-buffered saline (Gibco, Life Technologies) and ultracentrifuged again at 100,000× *g* for 60 min to eliminate any contaminating proteins. The microvesicles and exosomes were resuspended in 200 µL phosphate-buffered saline and stored at -80 °C.

Total RNA from LIM cells, cell-derived microvesicles, and cell-derived exosomes was isolated using mirVANA PARIS kits (Applied Biosystems, Thermo Fisher Scientific), according to the manufacturer instructions. MiRNA levels were measured using RT-qPCR with TaqMan miRNA assays (Applied Biosystems, Thermo Fisher Scientific). Ten nanograms of total RNA were reverse transcribed using TaqMan miRNA Reverse Transcription kits and miRNA-specific stem-loop primers (Applied Biosystems, Thermo Fisher Scientific) in a 15 µL reverse transcription reaction (composed of 0.15 µL 100 mM dNTPs mix, 1.5 µL 10× reverse transcription buffer, 0.19 µL RNase inhibitor [20 U/µL], 4.16 µL H_2O, 1 µL multiscribe reverse transcriptase, and 5 µL input RNA), using a TGradient thermal cycler (Biometra, Goettingen, Germany) at 16 °C for 30 min, 42 °C for 30 min, and 85 °C for 5 min. Real-time PCR was performed on the 5'-extended cDNA with TaqMan 2× Universal PCR Master Mix and the appropriate TaqMan MicroRNA Assay Mix for each miRNA of interest. Briefly, 4.5 µL 2.5-fold diluted reverse-transcribed product was combined with 5.5 µL PCR assay reagents to generate a PCR volume of 10 µL. Real-time PCR was carried out on a C1000 thermal cycler (CFX96 Real-Time system; Bio-Rad) at 95 °C for 10 min, followed by 40 cycles at 95 °C for 15 s and 60 °C for 1 min. The data were analyzed with CFX Manager software version V1.5.534.0511 (Bio-Rad). MiR-16 was used as an endogenous control for normalization. Normalized expression was calculated using the comparative Ct method, and fold-changes were derived from the $2^{-\Delta\Delta Ct}$ values for each miRNA.

d) Statistical tests

The local statistical tests aim at answering the following question: how significant is it to get that many number of hits in a given cluster/family? It can only be applied for networks in which there are clearly separated families/clusters. Keeping the same notations as in the main text, the p-value is provided by the following R function[11]:

1 - pbinom(nb_hits_cluster – 1, n_cluster, p),

with the proportion *p* of hits in the whole network *p* = n_hits / n_nodes, with n_hits the number of hits, and n_nodes the number of nodes with miRNA measurement in the network,
with *n_cluster* the size of the considered cluster/family,
with *nb_hits_cluster* the number of hits in the considered cluster/family.

The global statistical tests aim at answering the following questions: how significant miRNA hits tend to aggregate in a given network? It can be applied to all the networks. Due to the relative complexity of the networks, there is no simple formula. To calculate a p-value, we first estimate the distribution of the number hit pairs (edges connecting two miRNA hit nodes) under the null hypothesis H_0 by randomizing the hit tag on the nodes. For Seed2_7 network it provides near Gaussian, slightly positively

skewed curves, distributions. We generated 10^6 randomizations in each case to have a proper estimation of the distribution. The p-value is defined as the number of cases in H_0 the number of hit pairs is equal or greater than the number of hit pairs in the real case.

5. Step-by-step procedure to reproduce the Figures

Each Figure of the present study can be reproduced easily. First, the corresponding dataset (**Supplementary Tables 1-5**) has to be loaded into the webserver (main menu top left, click on 'Load data'). The downloadable file called 'mirviz-help.pdf' (top right) describes in detail the procedure in a dedicated section.

a) Figure 1: Differential expression and miRNA families

1. **Load** the 'STable1_Sequencing_Exosome_SRA106214.csv' file into miRViz.
    a. MiRNA column should be 'MIMAT'.
    b. Data column should be the last four ('LFC…' and 'Max_Expr').
2. Back in the 'Network' menu, **select the STable1** and LFC_C_vs_A33 in the 'Colors' section.
    a. Select the four-color scale to color the nodes. For differential expression, we often advise to set-up for each of the four colors: -2 / -0.5 / 0.5 / 2, and to keep the default colors. If so, strongly repressed miRNAs (LFC <-2) will be associated with a pure green color, moderately repressed miRNAs with a gradient of green (LFC -2 to -0.5), little to no differential expression will be white (LFC -0.5 to 0.5), moderately overexpressed miRNAs with a red gradient (LFC 0.5 to 2), and strongly overexpressed miRNAs pure red (LFC >2).
    b. Grey nodes correspond to unavailable data (miRNAs with no measurement).
3. **Add a new graph** in a second window (the color of the left band will change according to the window selected).
    a. Repeat the same procedure as in section 2 above for the LFC_C_vs_EpCam dataset.
    b. In a third window, repeat the procedure for the LFC_C_vs_sMV dataset.
    c. Three windows should be obtained, with three different datasets to be compared.
    d. Each window can be selected by the 'select' button below, or by zooming or moving the network inside a given window.
4. **Hide** miRNAs with very low expression.
    a. Poorly expressed miRNAs with differential expression are often affected by experimental noise. We suggest to hide them to focus on more interesting miRNAs.
    b. Click 'Display/Hide' in the top left menu. Select 'Max_Expr' from the data column. Select 5 and 21.24 (button pushed to the right), so that only miRNAs with a log2 expression of ≥5 under at least one of the conditions will be displayed.
    c. In Supplementary Figure 1A-C, we chose the semi-transparent option to show the underlying network. When looking for interesting areas, we suggest the 'transparent' option.
    d. Alternatively, for differential expression, transparency can be set-up with a p-value (only miRNAs associated with a low enough p-value will appear).

5. Navigate the networks to identify groups of interesting miRNAs and compare the datasets.
   a. The top right button 'Synchronize zoom for all networks allows navigation in the three windows in parallel. Click and move for displacement, and roll the mouse to zoom in or out. Pre-defined zoom levels are also proposed.
   b. Zooming in lets the miRNA name appear near the nodes. Alternatively, the 'Zoom dependent' button in the left band can be unclicked, and press 's' to show or remove the miRNA legend.
   c. A high resolution screen is highly recommended to benefit from miRViz. To further increase the size, we recommend full screen size of the browser (F11 or Control+Cmd+F, in most browsers), and to hide the left band of miRViz (double yellow arrows, top left).
   d. To scroll down the whole website, the mouse pointer should be located between two windows (to avoid zooming inside a window).
   e. The web browser can be zoomed out to see all three windows at once (often ctrl + roll, with the mouse pointer between two windows).

b) Figure 2: Survival data and genomic positions

The browser can be refreshed to start a completely new analysis. Alternatively, new data can be added to the previous analysis, or a new browser tab or window can be launched. A lot of loaded data slows down miRViz, as also for many tabs in the browser.

1. Load the 'STable2_Survival_AdrenocorticalCarcinoma_GSE49279.csv' file into miRViz.
   a. MiRNA column should be 'miR'.
   b. Data column should be the last two ('Median_expression' and 'log10_OS_sign').
   c. A few rows are ignored as they were not identified with miRViz nodes. Conversion to MIMAT identifiers would minimize the issue. MiRNAs removed from miRBase version 22 or earlier will also not be identified.

2. Change the **network.**
   a. Select the 'Genomic_Distance_50k' network.
   b. Click the 'One column' button, top right (near the 'Zoom' and 'Tool tip' buttons), to enlarge the window of interest.

3. Back in the 'Network' menu, **select the STable2** and log10_OS_sign in the section 'Colors'.
   a. To display p-values, we recommend the log scale. Here, a sign is added in the dataset to separate bad *versus* good prognosis miRNAs. MiRNAs with good prognosis have a negative sign.
   b. Select the four-color scale to color the nodes. Set up: -3 / -1 / 1 / 3, and keep the default colors. MiRNAs associated with OS with a p-value $<10^{-3}$ will be in pure color, as green for good prognosis, and red for bad prognosis. MiRNAs with a p-value of ≥0.1 will be pure white. MiRNAs with intermediate p-value will have a shaded color, as green or red, depending on the prognosis.
   c. Grey nodes correspond to unavailable data (miRNAs with no measurement).

4. Hide miRNAs with very low expression.

a. Poorly expressed miRNAs in most of the patients are often affected by experimental noise. We suggest to hide these to focus on more interesting miRNAs.
b. Click ‘Display/Hide’ in the top left menu. Select ‘Median_expression’ from the data column. Select 10 and 1609091 (button pushed to the right), so that only miRNAs with sufficient expression in at least half of the patients will be displayed.
c. In Supplementary Figure 2, we chose the semi-transparent option to show the underlying network. When looking for interesting areas, we suggest the ‘transparent’ option.

5. Navigation advice is the same as the previous case with differential expression.

c) Figure 3A: Differential expression and Diana50

1. Load the ‘STable3_MicroArray_DE_totipotent_GSE14473.csv’ file in miRViz.
a. MiRNA column should be ‘miR’.
b. Data column should be the last one (‘Median_LFC’).
c. A few rows are ignored as they were not identified with miRViz nodes. Conversion to MIMAT identifiers would minimize the issue. MiRNAs removed from miRBase version 22 or earlier will also not be identified.

2. Select the **Diana50 network**.

3. Back in the ‘Network’ menu, **select the STable3** and Median_LFC in the ‘Colors’ section.
a. Select the four-color scale to color the nodes. Set up: -2 / -0.5 / 0.5 / 2 (same as for case 1).
b. Grey nodes correspond to unavailable data (miRNAs with no measurement).

4. Hide miRNAs not measured.
a. Click ‘Display/Hide’ in the top left menu. Select ‘Median_LFC’ from the data column. Push both buttons respectively to the left and right.
b. In Supplementary Figure 3, we chose the semi-transparent option to show the underlying network. When looking for interesting areas, we suggest the ‘transparent’ option.

5. Navigation advice is the same as in case 1.

d) Figure 3B: Functional score and Diana50

1. Load the ‘STable4_Screening_bCSC_proportion.csv’ file into miRViz.
a. MiRNA column should be ‘MIMAT’.
b. Data column should be the last one (‘LFC’).
c. A few rows are ignored as they were not identified with miRViz nodes. This is due to the removal of these identifiers in miRBase v22 or earlier.

2. Select the **Diana50 network**.

3. Back in the ‘Network’ menu, **select the STable4** and LFC in the ‘Colors’ section.
a. Select the four-color scale to color the nodes. Set up: -3 / -1 / 1 / 3 (scale to highlight stronger effects as compared to the scale chosen in cases 1 and 3).
b. Grey nodes correspond to unavailable data (miRNAs with no measurement).

4. Hide miRNAs not measured
a. Click ‘Display/Hide’ in the top left menu. Select ‘LFC’ from the data column. Push both buttons respectively to the left and right.

b. In Supplementary Figure 3, we chose the semi-transparent option to show the underlying network. When looking for interesting areas, we suggest the 'transparent' option.

5. Navigation advice is the same as in case 1.

e) Figure 4 and Supplementary Figure 2: Ontology enrichment and co-regulation networks

1. Load the 'STable5_GOenrichment_mRNA_targets.csv' file into miRViz.
   a. MiRNA column should be 'MIMAT'.
   b. Data column should be the last two ('Reg. gene expr.' and 'small GTPase mediated sign. transd.').

2. Select the **Diana50 (Figure 4)** or the **TargetScan54 (Supplementary Figure 2) network**.

3. Back in the 'Network' menu, **select the STable5** and 'Reg. gene expr.' in the section 'Colors'.
   a. Select the **three-color scale** to color the nodes. Set up: Unchanged / 5 / 10. The first color should be turned to white. Thus, the miRNA nodes with p-values $>10^{-5}$ are in white, those between 10^{-5} and 10^{-10} are red shaded, and those $<10^{-10}$ are red.

4. Add a new graph in a second window (the color of the left band will change according to the selected window).
   a. Repeat the same procedure as section 3 above for the 'small GTPase mediated sign. transd.' Dataset.

5. Navigation advice is the same as in case 1.

f) Generating high-quality images

Typically, three possibilities are proposed to generate high-quality images of the colored miRNA networks:

1. Intermediate quality, but easy option.
   a. Export as png (export button below each window), and assemble the figure.
   b. Higher resolution images are obtained from higher resolution screens.
2. High quality images option.
   a. Export as pdf – the image is a scalable vector graphic, so that it can be zoomed in without quality loss.
   b. This solution requires high RAM memory, especially with the Genomic_Distance and TargetScan54 networks.
   c. Assemble the figures with vector drawing software (such as Inkscape or CorelDraw).
3. High quality and more flexible option.
   a. Download the cytoscape[8] file through the 'Download file' icon, top right.
   b. Benefit from the flexibility of Cytoscape to adapt your drawings. For example, multiple values can be displayed on each node using the enhancedGraphics plugin[21].

6. Statistical tests results

a) Local statistical tests

The local p-value is provided in each case in the table below, corresponding to Figure 1.

Proportion of hits / Name and size of the family	**A33**	**EpCam**	**sMV**
P (proportion of hits, LFC > 1)	0.18 (28 hits/156 nodes)	0.13 (20/156)	0.058 (9/156)
320 family (5 expressed nodes)	5 hits pvalue = $1.9\ 10^{-4}$	5 hits pvalue = $3.5\ 10^{-5}$	5 hits pvalue = $1.9\ 10^{-4}$
98/let-7 family (5 expressed nodes)	5 hits pvalue = $1.9\ 10^{-4}$	5 hits pvalue = $3.5\ 10^{-5}$	5 hits pvalue = $6.4\ 10^{-7}$
378/422a family (9 expressed nodes)	6 hits pvalue = $1.7\ 10^{-3}$	0 hit pvalue = 1	0 hit pvalue = 1

b) Global statistical tests

The global p-value is provided in each case in the table below, corresponding to Figure 1:

Characteristics	**A33**	**EpCam**	**sMV**
Number of hits	28	20	9
Number of hit pairs	41	25	12
H0 (Mean, median, standard deviation, skewness)	μ = 9.8 median = 9.0 σ = 3.4 skew = 0.84	μ = 4.7 median = 4.0 σ = 2.3 skew = 1.0	μ = 1.2 median = 1.0 σ = 1.1 skew = 1.4
p-value	$1\ 10^{-6}$	$5\ 10^{-6}$	$8\ 10^{-6}$

References

1. Kozomara, A., Birgaoanu, M. & Griffiths-Jones, S. miRBase: from microRNA sequences to function. *Nucleic Acids Res.* **47**, 155–162 (2018).
2. Bartel, D. P. Metazoan MicroRNAs. *Cell* **173**, 20–51 (2018).
3. Chang, T. C., Pertea, M., Lee, S., Salzberg, S. L. & Mendell, J. T. Genome-wide annotation of microRNA primary transcript structures reveals novel regulatory mechanisms. *Genome Res.* **25**, 1401–1409 (2015).
4. Flor, I. & Bullerdiek, J. The dark side of a success story: microRNAs of the C19MC cluster in human tumours. *J. Pathol.* **227**, 270–274 (2012).
5. Maragkakis, M. *et al.* DIANA-microT web server: Elucidating microRNA functions through target prediction. *Nucleic Acids Res.* **37**, 273–276 (2009).
6. Lewis, B. P., Burge, C. B. & Bartel, D. P. Conserved seed pairing, often flanked by adenosines, indicates that thousands of human genes are microRNA targets. *Cell* **120**, 15–20 (2005).
7. Bhajun, R. *et al.* A statistically inferred microRNA network identifies breast cancer target miR-940 as an actin cytoskeleton regulator. *Sci. Rep.* **5**, 8336 (2015).
8. Shannon, P. *et al.* Cytoscape: A software Environment for integrated models of biomolecular interaction networks. *Genome Res.* **13**, 2498–2504 (2003).
9. Pinzón, N. *et al.* MicroRNA target prediction programs predict many false positives. *Genome Res.* **27**, 234–245 (2017).
10. Karagkouni, D. *et al.* DIANA-TarBase v8: A decade-long collection of experimentally supported miRNA-gene interactions. *Nucleic Acids Res.* **46**, D239–D245 (2018).
11. Team, R. C. R: A language and environment for statistical computing. (2017).
12. Ji, H. *et al.* Deep sequencing of RNA from three different extracellular vesicle (EV) subtypes released from the human LIM1863 colon cancer cell line uncovers distinct mirna-enrichment signatures. *PLoS One* **9**, (2014).
13. Langmead, B., Trapnell, C., Pop, M. & Salzberg, S. L. Ultrafast and memory-efficient alignment of short DNA sequences to the human genome. *Genome Biol.* **10**, (2009).
14. Love, M. I., Huber, W. & Anders, S. Moderated estimation of fold change and dispersion for RNA-seq data with DESeq2. *Genome Biol.* **15**, 550 (2014).
15. Assié, G. *et al.* Integrated genomic characterization of adrenocortical carcinoma. *Nat. Genet.* **46**, 607–612 (2014).
16. Stadler, B. *et al.* Characterization of microRNAs Involved in Embryonic Stem Cell States. *Stem Cells Dev.* **19**, 935–950 (2010).
17. El Helou, R. *et al.* miR-600 Acts as a Bimodal Switch that Regulates Breast Cancer Stem Cell Fate through WNT Signaling. *Cell Rep.* **18**, 2256–2268 (2017).
18. Agarwal, V., Bell, G. W., Nam, J. W. & Bartel, D. P. Predicting effective microRNA target sites in mammalian mRNAs. *Elife* **4**, 1–38 (2015).
19. Carlson, M., Falcon, S., Pages, H. & Li, N. GO.db: A set of annotation maps describing the entire Gene Ontology. R package version 3.1.2.
20. Durinck, S., Spellman, P. T., Birney, E. & Huber, W. Mapping identifiers for the integration of genomic datasets with the R/Bioconductor package biomaRt. *Nat. Protoc.* **4**, 1184–91 (2009).
21. Morris, J. H., Kuchinsky, A., Ferrin, T. E. & Pico, A. R. EnhancedGraphics: A Cytoscape app for enhanced node graphics. *F1000Research* **3**, 1–9 (2014).

Legends for Supplementary Figures

Supplementary Figure 1. Experimental validation of the selective enrichment of the miR-320 family in exosomes (corresponding to the red squares in **Figure 1**) using RT-qPCR.

Supplementary Figure 2: Gene ontology enrichment for predicted targets of individual miRNAs overlaid on top of the TargetScan54 network. Red nodes correspond to miRNAs predicted to regulate many protein coding genes known to participate in the following ontologies: (**A**) GO:0010468 (regulation of gene expression); (**B**) GO:0007264 (small-GTPase-mediated signal transduction). This is the same as for **Figure 4**, but it uses the TargetScan54 network.
