# Supplementary figures and images for: miRViz: a novel webserver application to visualize and interpret microRNA datasets
Source: Nucleic Acids Res. 2020 Apr 22;48(W1):W252–61. doi: 10.1093/nar/gkaa259 (PMC7319447; doi:10.1093/nar/gkaa259)

Supplementary Figure 1

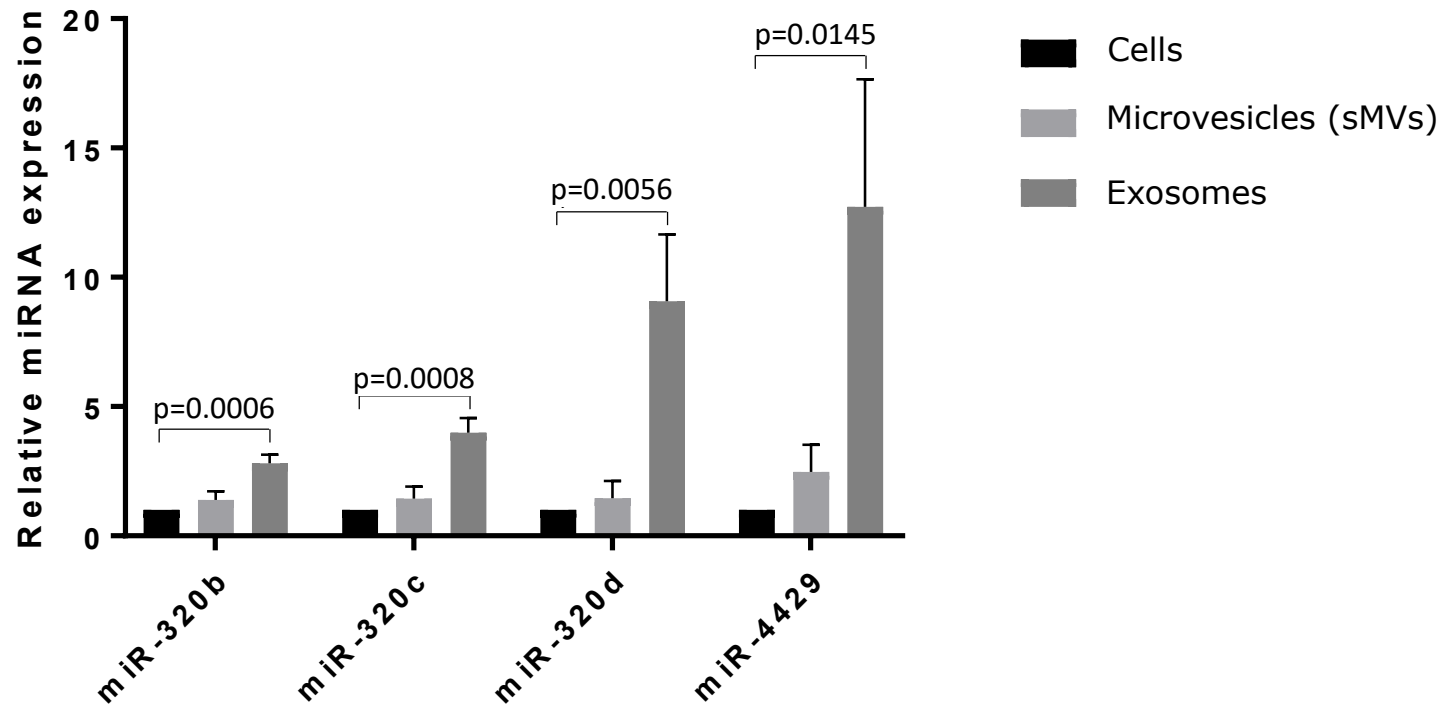

Supplementary Figure 2A

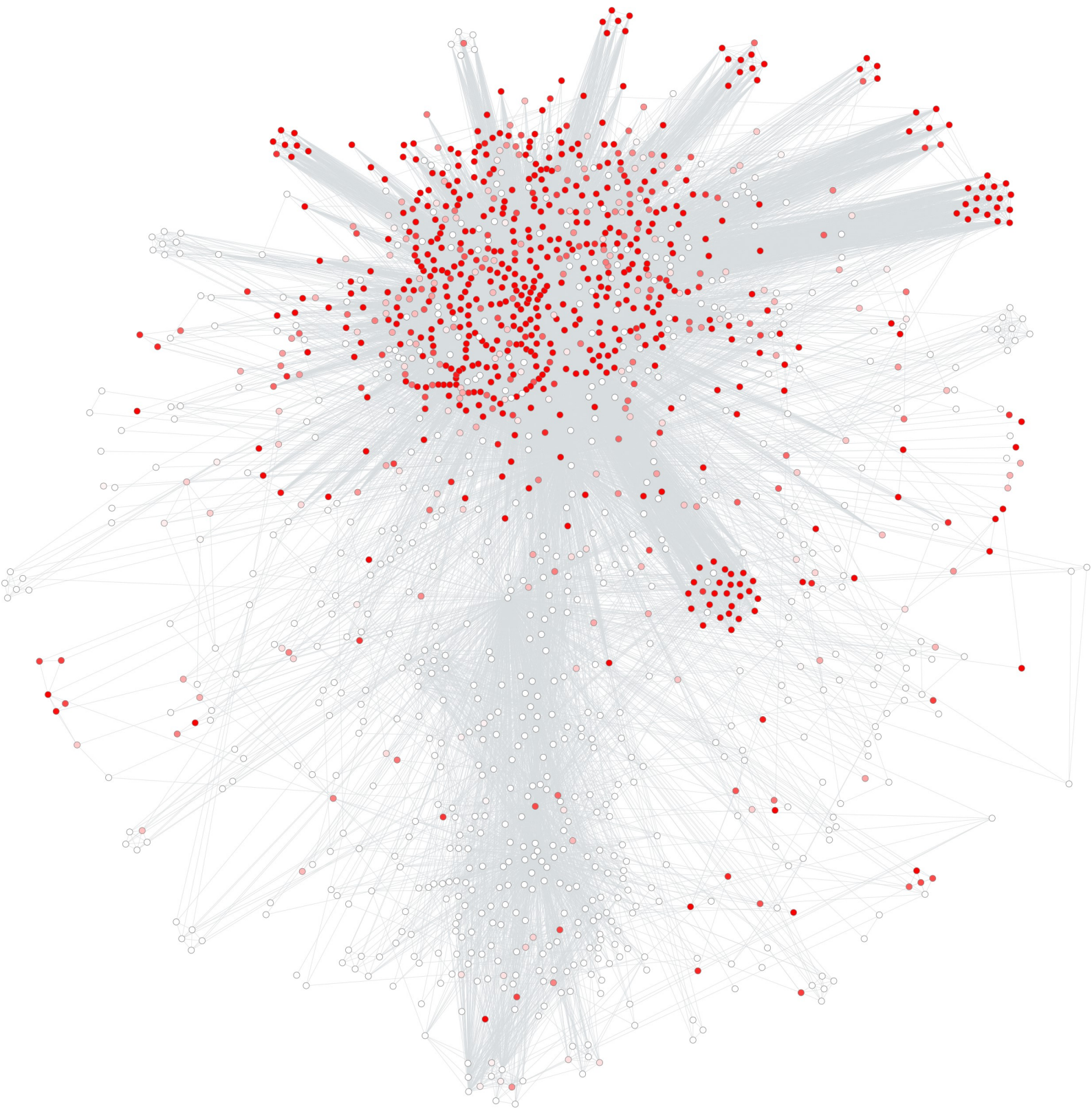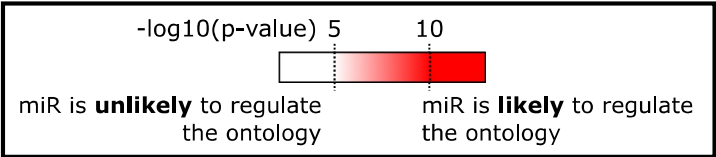

Supplementary Figure 2B

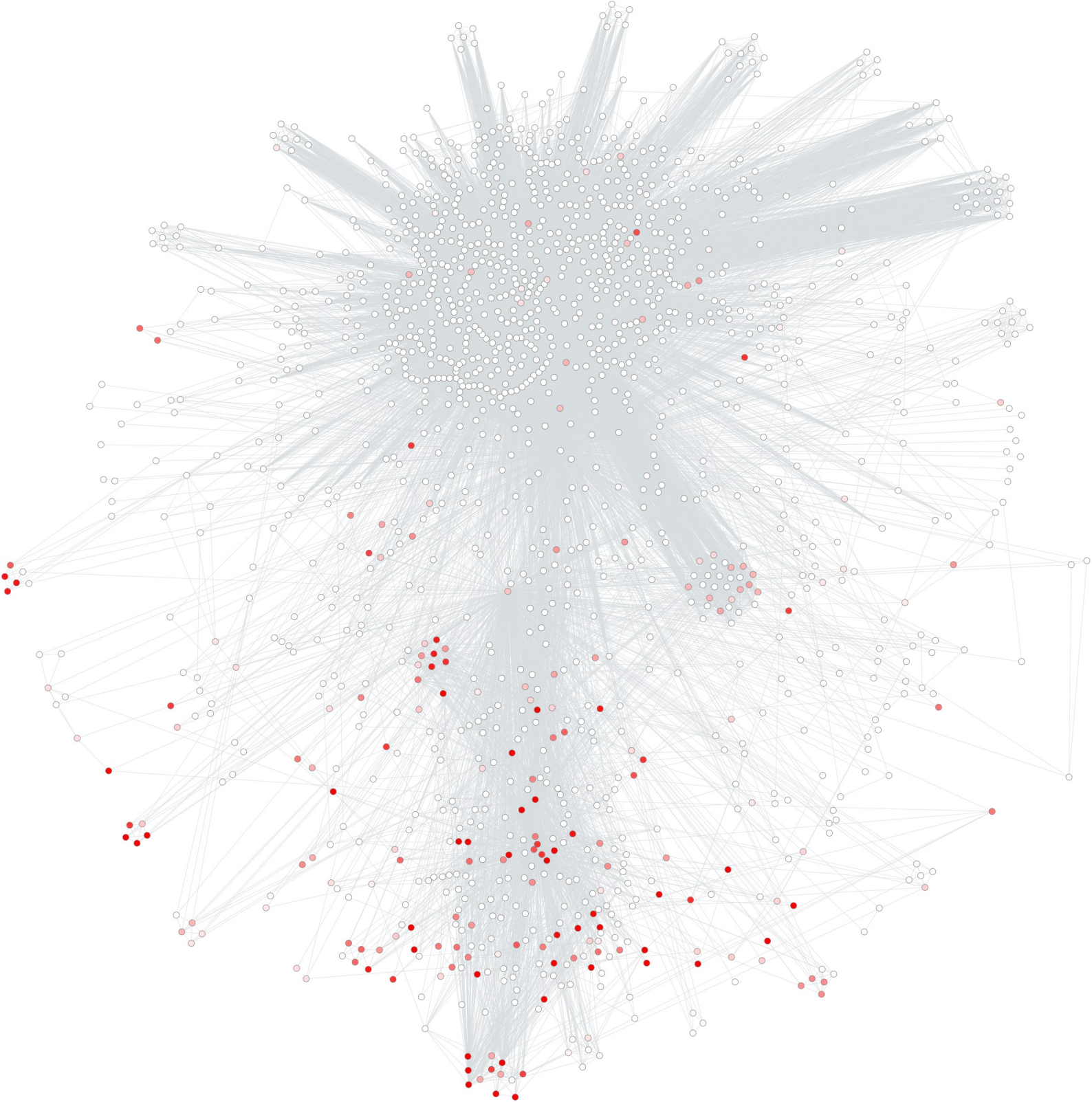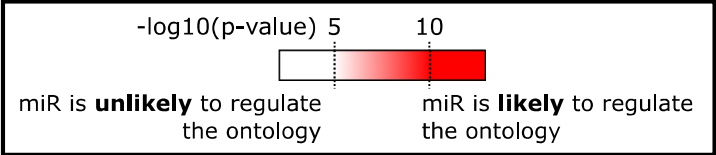

Supplement: gkaa259_Supplemental_Files [file gkaa259_supplemental_files.zip › SupplementaryFigures.pdf]
